# Variation in Inflammatory Cytokine/Growth-Factor Genes and Mammographic Density in Premenopausal Women Aged 50–55

**DOI:** 10.1371/journal.pone.0065313

**Published:** 2013-06-07

**Authors:** Ali Ozhand, Eunjung Lee, Anna H. Wu, Merete Ellingjord-Dale, Lars A. Akslen, Roberta McKean-Cowdin, Giske Ursin

**Affiliations:** 1 Department of Preventive Medicine, Norris Comprehensive Cancer Center, University of Southern California Keck School of Medicine, Los Angeles, California, United States of America; 2 Institute of Basic Medical Sciences, University of Oslo, Oslo, Norway; 3 Centre for Cancer Biomarkers, The Gade Laboratorium for Pathology, Department of Clinical Medicine, University of Bergen, Bergen, Norway; 4 Cancer Registry of Norway, Oslo, Norway; MOE Key Laboratory of Environment and Health, School of Public Health, Tongji Medical College, Huazhong University of Science and Technology, China

## Abstract

**Background:**

Mammographic density (MD) has been found to be an independent risk factor for breast cancer. Although data from twin studies suggest that MD has a strong genetic component, the exact genes involved remain to be identified. Alterations in stromal composition and the number of epithelial cells are the most predominant histopathological determinants of mammographic density. Interactions between the breast stroma and epithelium are critically important in the maturation and development of the mammary gland and the cross-talk between these cells are mediated by paracrine growth factors and cytokines. The potential impact of genetic variation in growth factors and cytokines on MD is largely unknown.

**Methods:**

We investigated the association between 89 single nucleotide polymorphisms (SNPs) in 7 cytokine/growth-factor genes (*FGFR2, IGFBP1, IGFBP3, TGFB1, TNF*, *VEGF*, *IL6*) and percent MD in 301 premenopausal women (aged 50 to 55 years) participating in the Norwegian Breast Cancer Screening Program. We evaluated the suggestive associations in 216 premenopausal Singapore Chinese Women of the same age.

**Results:**

We found statistically significant associations between 9 tagging SNPs in the *IL6* gene and MD in Norwegian women; the effect ranged from 3–5% in MD per variant allele (p-values = 0.02 to 0.0002). One SNP in the *IL6* (rs10242595) significantly influenced MD in Singapore Chinese women.

**Conclusion:**

Genetic variations in *IL6* may be associated with MD and therefore may be an indicator of breast cancer risk in premenopausal women.

## Introduction

High mammographic density (MD) is an established risk factor for breast cancer. Women with extensive MD have been found to have four to six times the risk of breast cancer compared to women with little or no density [1]–[3]. MD is influenced by several breast cancer risk factors including age, body mass index (BMI), parity, age at first birth, hormone therapy use and physical activity; these variables jointly explain approximately 30% of the variability in MD [4]. It is likely that genetic variation is another key factor influencing variability in MD. Twin studies suggest that genetic factors account for 30–60% of the variance in MD [5]–[7]. However, the genetic determinants of MD have not yet been identified. In a recent combined meta-analysis of data from five genome wide association studies (GWAS) among women of European descent, one locus (*ZNF365-* rs10995190) was reported as highly associated with MD after correction for age and BMI. Although highly statistically significant (combined P = 9×6·10−^10^), this SNP explains only 0.5% of the variance in MD [8]. It seems likely that there will be multiple other loci involved not detected in the GWAS given the low statistical power of GWAS [9], [10].

The histopathological composition of dense breast tissue consists of both stroma and concentrated epithelial tissue [11]. Mammographically dense breasts have been shown to have higher amounts of collagen, more extensive stromal fibrosis, and higher numbers of epithelial cells when compared with breasts with little density [11]–[14]. Breast stroma and epithelium interact by means of paracrine cytokines and growth factors, which is a necessary process in the normal maturation and development of the mammary gland [15]–[17].

The stroma includes fibrous connective tissue, extracellular matrix (ECM) proteins, fibroblasts, adipocytes, endothelial cells, and innate immune cells. Stroma provides physical structure for the gland and stromal cells secrete signals that are important in the development and function of the epithelium [18]. The extracellular matrix (ECM) together with growth factors/cytokines and cell-cell interactions, modulate the shape, polarity and behavior (survival, proliferation, differentiation, or migration) of cells in mammary tissue [19]. The interactions between cells and ECM are also crucial in determining the organization of the ECM itself [20], [21]. Both cell behavior and tissue structure is therefore affected by cell-ECM interactions. Thus, studying the growth factor/cytokines, as the important signals in the mammary tissue microenvironment, and their role in determining mammographic density, as a marker of the tissue structure and breast cancer, is crucial for understanding mechanisms of breast cancer development.

A number of studies have suggested an association between growth factors and cytokines and MD. Specifically, serum levels of IGF-I and IGF binding proteins have been associated with MD [22]–[24]; findings have been more consistent in premenopausal than in postmenopausal women [24]–[26]. Further, quantitative microscopy using immunoreactive staining has shown higher amounts of IGF-I in dense breasts compared with lower density breasts, especially in women younger than 50 years of age [13]. Genetic variations in IGF and IGF binding proteins have been associated with MD in several studies [7], [27]–[30]. The role of other growth factors and cytokines such as transforming growth factor-beta (TGF-β), interleukins and tumor-necrosis-factor-alpha (TNF-α) with MD has not been well described. A gene expression analysis found decreased levels of TGF-β signaling in women with increased MD [31]. One study observed a positive association between serum levels of interleukin-6, TNF-α, and C-reactive protein (CRP) with MD. Although that association did not remain statistically significant after adjusting for BMI [32], the sum of the findings to date was supportive, and we decided to further study the association between growth factor genetic variants and MD.

Given the biological constituents of MD, the known role of hormone therapy on MD [33], [34], and the individual variability in such hormonal effects, we recently investigated the association between genetic variants in 23 hormone metabolism genes and 7 growth factor genes and MD in postmenopausal participants of the Norwegian Breast Cancer Screening Program (NBCSP). That analysis suggested that there was an association with genetic variants in *PRL* and *CYP1B1* in hormone users (most of whom had used norethisterone acetate preparations). In women who had never used hormone therapy, it was not a hormone gene, but a growth factor gene that was most important (genetic variants in *TNF-α*.) This suggests that genetic determinants of MD may vary depending on women’s hormonal milieu, and indicated that in never users of hormone therapy growth factor genes may play a role [35].

We therefore explored the role of variation in growth factor genes in premenopausal women participating in NBCSP and compared the results with our previous findings in postmenopausal women. We also decided to test any association in an independent sample of similarly aged premenopausal Singapore Chinese women.

## Materials and Methods

### Study Population: Norwegian Breast Cancer Screening Program (NBCSP) Participants

The NBCSP is a governmentally funded program which provides biennial screening mammograms to all Norwegian women 50–69 years of age. The screening program began as a four-year pilot project in 1995–96 in four counties of Norway. The project was expanded to all 19 counties and became a nationwide program in 2004. As part of the NBCSP, all women of the appropriate age are sent an invitation letter to receive a bilateral two-view mammogram biennially. Each woman is given an appointment time and location for receiving the mammogram. During the first 10 years (1996–2005), 76.2% of invited women participated in the screening program [36].

In 2004, 17,050 female residents of the three largest counties in Norway (Oslo, Akershus, and Hordaland) were invited to participate in the current study at the same time as they were mailed the official NBCSP invitation letter. This study has previously been described [37]. In brief, participants were asked to complete a risk factor questionnaire which included questions on menstrual and reproductive history, oral contraceptive and menopausal hormone use, family history of breast cancer, current weight and height, alcohol and smoking. Subjects were asked to bring the completed questionnaire and informed consent to the clinic on the day of their scheduled mammogram. Approximately 71% (N = 12,056) of the invited women attended the scheduled mammographic examination and 66% of the attendees aged 50 to 69 (N = 7,941) completed the risk factor questionnaire.

Buccal kits were mailed to 7,174 of the 7,941 women who completed the mammogram and questionnaire to collect DNA for genetic testing. A total of 3,728 women (51% of the 7,174 women) provided a buccal sample. We requested mammograms from the radiological facilities on all 3,728 women with a completed questionnaire and a buccal sample. After excluding women with only a digital mammogram (n = 300), we were able to obtain analog mammograms from the year 2004 on 2,876 women. Of these, 121 women were excluded for the following reasons; history of breast or any cancers (N = 17), undetermined breast area (N = 3), missing age (N = 28), missing BMI (N = 73) (height = 46/weight = 67). After the exclusions, a total of 2,755 women aged 50 to 69 had usable analog mammogram and complete risk factor data. All the participants signed an informed consent and the study was approved by the USC institutional review board, the Norwegian regional ethics committee and the Norwegian Data Inspectorate.

### Mammographic Density Assessment

Left craniocaudal mammograms were scanned using a Kodak Lumisys 85 scanner. MD was assessed by a trained reader (GU) using a previously validated computer-assisted method (the University of Southern California Madena software) [38] **.** The reader assessed the absolute MD by outlining all dense areas within the breast except white artifacts, prominent fibrous strands, vasculature or the pectoralis muscle. The total area of the breast was assessed by a research assistant who was trained by GU. MD was calculated as the absolute density divided by the total area of the breast.

### Tagging SNP Selection and Genotyping

We selected genes encoding growth factors (*VEGF*), growth factor receptors (*FGFR2, GHRHR*), growth factor binding proteins (*IGFBP1; IGFBP3*), and cytokines (*TGFB1, TNF, IL6*). For *VEGF, IGFBP1;IGFBP3, TGFB1, TNF*, and *IL6*, we selected tagging SNPs to capture the genetic variation in each gene with an R^2^>0.80. Tagging SNPs were selected from 20 kb upstream of 5′ untranslated region (UTR) to 10 kb downstream of 3′ UTR that tagged all common SNPs (minor allele frequency ≥5%) among the non-Hispanic white or Chinese population. This selection was done using the Snagger [39] software and a custom database of the Hapmap CEU data (http://hapmap.ncbi.nlm.nih.gov); release 24) merged with the Affymetrix 500 K panel as well as the Hapmap CHB data release 24. For *FGFR2* and *GHRHR*, we selected one SNP of interest for each gene.

Due to restricted funding, DNA extraction and genotyping were performed on 3,317 of the 3,728 participants who donated buccal samples. DNA was extracted from buccal swabs using the standard protocol for the QIAamp blood DNA kit (Qiagen, Valencia, CA). We genotyped the selected SNPs using an Illumina BeadLab System (San Diego, CA) with GoldenGate®. Genotyping was completed in the USC Genomics Center under the direction of Dr. David Van Den Berg. Briefly, samples were run in a 96-well format using the Illumina Sentrix Array technology, scanned on a BeadArray Reader, and analyzed using BeadStudio Software (v.3.0.9) with Genotyping Module (v.3.0.27) (Illumina). The SNPs with <85% call rates were excluded: this resulted in the exclusion of 4% of SNPs. The genotyping concordance rate based on 57 duplicate samples was 98%. Out of 97 SNPs in this pathway, 8 SNPs were excluded due to departure from Hardy-Weinberg equilibrium (HWE) (P<0.001), leaving 89 SNPs for further analysis.

Of the genotyped 3,317 samples, 241 samples were excluded from the analysis due to low overall genotype call-rates (less than 80%). In total, 2,397 women (2,055 postmenopausal, 342 peri- or premenopausal at the time of mammography) had complete information on genotype, MD and breast cancer risk factors. Of the 342 peri- or premenopausal women, 301 were premenopausal and aged 55 or younger at the time of mammogram (they were still menstruating and were not taking any type of hormones).

### Statistical Analysis

We explored the association between MD and potential risk factors (age, BMI, age at full-term pregnancy, number of children, age at menarche, family history of breast cancer, and level of education) using categorical variables. We used analysis of covariance (ANCOVA) to calculate age adjusted least-square mean of MD in each category. A test of trend across these categories was generated using linear regression models after adjusting for age; BMI was further included in the models [40].

We investigated the association between each genetic variant and MD based on additive models, which estimate the difference in the continuous dependent variable (MD) per copy of the minor allele of each polymorphism after adjustments for age and BMI. In order to explore the potential modifying effect of BMI on the findings, we repeated this analysis separately in women with BMI below as well as above 25 kg/m^2^. We considered a two-sided *P* value of <0.05 as statistically significant.

### Replication Study and Combined Analysis

We evaluated the statistically significant associations observed in the NBCSP participants using data from 163 premenopausal Singapore Chinese women of similar age, who were participants of the genetic study component of the Mammography Subcohort of the Singapore Chinese Health Study (SCHS). Participants of the Mammography Subcohort were enrolled in both the SCHS and the Singapore Breast Screening Project (SBSP); details have been described previously [41], [42]. Briefly, 35,298 Chinese women and 27,959 men, ages 45–74 years, enrolled in SCHS during 1993–1998. Subjects were residents of government housing estates; during the enrollment period 86% of the Singapore population resided in such housing facilities. During 1994 to 1997, Singaporean women ages 50–64 years were invited for a screening mammography as part of the SBSP [43]. Through a computer linkage, a total of 3,777 women common to the SBSP and SCHS databases were identified. Of these, mammograms were successfully retrieved from 3,702 women. We excluded 6 women due to missing information on key variables; 1 woman who was later found not to be a Singapore resident. Mammograms of the Mammography Subcohort of the SCHS were scanned using a Cobrascan 812T scanner (Radiographic Digital Imaging Inc., Compton, California). Images were read using the same procedures and software by GU. The total breast area was assessed by two assistants and the average of the two readings was used. Of the 3,695 women in the Mammography Subcohort [41], [44], [45], DNA samples were available on 2,164 women (1,848 blood, 316 buccal). Twenty tagging SNPs in the *IL6* locus were selected and genotyped using the same methods used for the NBCSP participants; 1 SNP with a genotyping call rate <85% and 7 SNPs with a MAF<0.01 in Chinese population were excluded, leaving 12 *IL6* SNPs for statistical analyses. 2,038 samples of the 2,164 genotyped samples had a genotyping success rate (call rate ≥85%). The mean age of the 2,038 participants were 57.2 (SD 4.3). Two hundred and sixteen women self-reported as premenopausal at time of mammography; of these, 163 women who were aged 55 or younger at mammography (range 46–55) and had never used hormone therapy were included in the current analysis. Genotyping concordance based on the 42 random duplicate samples was >99.9%. None of the 12 *IL6* SNPs departed significantly from HWE (P≥0.01).

We combined the Norwegian and Singapore samples and assessed the association between 12 *IL6* tagging SNPs and MD. In the combined analysis, we defined the risk allele as the minor allele in the Norwegian sample. We adjusted the models for age at mammogram (continuous), BMI at mammogram (continuous), and ethnic and dialect group (Norwegian, Cantonese, Hokkien).

## Results

### Baseline Characteristics of the Participants

The baseline characteristics of the postmenopausal sample have previously been described [35]. In brief, mean age at screening was 58.4 years, mean BMI 25.1, mean age at menarche 13.2 years, mean age at first pregnancy 22.0 years, mean number of children 2.0, and mean years of education 12.8. In premenopausal women (Table 1), mean MD decreased with increasing BMI after adjustment for age (P<0.0001). Older age at full term pregnancy was associated with higher MD after adjustment for age and BMI (P = 0.02). Higher level of education was associated with higher percent MD after adjustment for age (P = 0.011) but the association was no longer statistically significant after we further adjusted the model for BMI.

**Table 1:** Mean percentage of mammographic density (MD) by descriptive characteristics (n = 301).

		N	%	%MD^a^	P^b^	%MD d	P
**Age (years)**	**50**	107	35.55	25.35		25.32	
	**51**	92	30.56	26.92		27.58	
	**52–55**	102	33.89	25.42	0.8516	24.86	0.8157
**BMI (Kg/m2)**	**<20**	18	5.98	44.78		34.04	
	**20–23**	95	31.56	32.44		26.01	
	**23–25**	67	22.26	26.32		24.74	
	**25–30**	88	29.24	20.04		24.26	
**Age at first full term pregnancy**	**>30**	33	10.96	11.13	<0.0001	27.44	0.1848
	** = <20**	46	15.97	19.45		19.64	
	**21–24**	78	27.08	24.32		25.21	
	**25–29**	72	25	29.94		28.3	
	**> = 30**	64	22.22	25.51	0.0364^c^	26.48	0.0223^c^
	**Nulliparous**	28	9.72	30.69	0.011	29.91	0.0079
**Number of children**	**Nulliparous**	28	9.3	30.63		29.76	
	**One**	46	15.28	26.22		28.67	
	**Two**	145	48.17	24.2		26.06	
	**Three or more**	82	27.24	26.94	0.4829	24.09	0.1787
**Age at menarche**	**Younger than 12**	38	12.62	22.46		26	
	**12**	68	22.59	25.25		26.17	
	**13**	88	29.24	26.01		25.31	
	**14**	59	19.6	25.28		24.96	
	**Older than 14**	48	15.95	29.8	0.1013	27.4	0.8585
**Family history of breast cancer**	**No**	218	72.43	26		25.6	
	**Yes**	83	27.57	25.48	0.8247	26.51	0.6621
**Level of education**	**Secondary & below**	49	17.25	19.93		24.24	
	**Higher secondary**	100	35.21	26.44		26.25	
	**College/university**	135	47.54	28.28	0.0102	26.86	0.3775

a Percent MD adjusted for age at mammogram (continuous).

b test for trend.

c test for trend excluding the nulliparous group.

d Percent MD adjusted for age at mammogram (continuous) and BMI at mammogram (continuous).

### Associations between SNPs and Mammographic Density in NBCSP Participants

The effect of growth factor gene variants on MD was significantly modified by menopausal status (Table S1; see [35] for detailed results on postmenopausal women). The majority of statistically significant associations were observed among the premenopausal women only. In the remaining part of the results, we limit the analysis to this group of women.

### Associations between SNPs and Mammographic Density in Premenopausal NBCSP Participants

In the additive genetic model, *IL6* tagging SNPs rs6952003, rs10242595, rs11766273, rs1880241, rs1880242, rs2069833, rs2069840, rs4552807 and rs7776857 were associated with MD with P values less than 0.05 (Table 2 and Table 3). The estimated difference in MD per minor allele of each *IL6* SNP ranged from 3–5%, with p-values ranging from 0.04 to 0.0002. One *TNF* tagging SNP (rs2857605) was also significantly associated with MD (beta = 2.99), however the level of significance was relatively low (P = 0.046). We did not find any statistically significant associations between the polymorphisms in *VEGF, GHRHR, IGFBP1, IGFBP3, FGFR2*, and *TGFB1* and MD (Table 2 and Table S2).

**Table 2:** The association between the most significant SNP within each growth factor gene and MD in Norwegian women (N = 310).

Gene name	Number of SNPs tested	Most significant SNP	WW^a^	WV^b^	VV^c^	MAF^d^	beta^e^	SE	P
**IL6**	18	rs1880241	85	158	54	0.45	4.98	1.34	0.0002
**TNF**	15	rs2857605	182	92	21	0.22	2.99	1.49	0.046
**VEGF**	19	rs3025030	217	78	3	0.13	3.51	1.98	0.07
**IGFBP1;IGFBP3**	26	rs13232606	261	25	1	0.05	4.91	3.10	0.12
**TGFB1**	9	rs12983047	209	70	16	0.16	1.04	1.62	0.52
**GHRHR**	1	rs4988496	263	25	0	0.05	−4.33	3.36	0.20
**FGFR2**	1	rs2981582	113	139	42	0.36	0.78	1.36	0.57

a Number of women with wild-wild genotype.

b Number of women with wild-variant genotype.

c Number of women with variant-variant genotype.

d Minor allele frequency.

e Percent MD per variant allele based on additive model adjusted for age at mammogram (continuous) and BMI at mammogram (continuous).

**Table 3:** Association between 9 IL6 tagging SNPs (with P-value less than 0.05) and MD after adjustment for age and BMI, based on an additive genetic model (N = 301).

Gene name	SNP	Chromosome Position^a^	Alleles	WW^b^	WV^c^	VV^d^	MAF^e^	beta^f^	SE	P
IL6	rs1880241	22759469	A:G	85	158	54	0.45	4.98	1.34	0.0002
IL6	rs10242595	22774231	G:A	154	125	15	0.25	5.60	1.56	0.0004
IL6	rs7776857	22754768	T:G	110	150	37	0.38	−4.87	1.39	0.0005
IL6	rs2069833	22767664	T:C	81	157	56	0.47	−4.61	1.36	0.0008
IL6	rs1880242	22759607	G:T	70	162	66	0.46	3.84	1.37	0.0053
IL6	rs4552807	22751019	T:A	110	146	39	0.42	3.54	1.37	0.010
IL6	rs11766273	22775663	G:A	247	48	1	0.08	−5.84	2.40	0.015
IL6	rs2069840	22768572	C:G	132	133	31	0.31	3.40	1.41	0.016
IL6	rs6952003	22752705	T:A	173	113	10	0.23	3.44	1.66	0.0399

a based on map to Genome Build 37.3.

b Number of women with wild-wild genotype.

c Number of women with wild-variant genotype.

d Number of women with variant-variant genotype.

e Minor allele frequency.

f Percent MD per variant allele based on additive model adjusted for age at mammogram (continuous) and BMI at mammogram (continuous).

In addition, we examined the associations separately in women with low and high BMI (using 25 kg/m^2^ as the cut-off value). The association between *IL6* SNPs and MD appeared to be restricted to women with a BMI less than 25 kg/m^2^; 8 of 9 tagging *IL6* SNPs that showed significant results in the overall analysis remained significant only in the low BMI group. For 5 of these 8 SNPs, the effect modification by BMI was statistically significant (Table 4).

**Table 4:** Association between 8 IL6 tagging SNPs from table 3 and MD in low and high BMI groups.

		BMI< = 25 n = 180						BMI>25 n = 121						
gene name	SNP	beta^a^	SE	P	WW^b^	WV^c^	VV^d^	beta^a^	SE	P	WW	WV	VV	P^e^
**IL6**	**rs10242595**	8.66	2.04	<.0001	101	67	10	1.47	2.28	0.52	53	58	5	0.04
**IL6**	**rs2069833**	−7.71	1.80	<.0001	48	93	35	−1.17	1.96	0.55	33	64	21	0.07
**IL6**	**rs7776857**	−7.57	1.81	<.0001	68	85	24	−1.75	2.08	0.40	42	65	13	0.72
**IL6**	**rs4552807**	6.32	1.82	0.0007	70	85	22	−0.21	1.99	0.92	40	61	17	0.06
**IL6**	**rs1880242**	6.07	1.79	0.0009	41	92	45	1.31	2.04	0.52	29	70	21	0.02
**IL6**	**rs11766273**	−10.00	3.13	0.0017	142	34	0	−2.38	3.69	0.52	105	14	1	0.88
**IL6**	**rs1880241**	5.60	1.86	0.0029	50	96	32	4.72	1.83	0.0112	35	62	22	0.01
**IL6**	**rs6952003**	5.97	2.27	0.0091	104	67	6	−0.41	2.33	0.86	69	46	4	0.03
**IL6**	**rs2069840**	3.69	1.90	0.054	71	85	21	3.44	2.00	0.09	61	48	10	0.03

a Percent MD per variant allele based on additive model adjusted for age at mammogram (continuous) and BMI at mammogram (continuous).

b Number of women with wild-wild genotype.

c Number of women with wild-variant genotype.

d Number of women with variant-variant genotype.

e P-value for interaction.

### Association between *IL6* SNPs and MD in Singapore Chinese Women

Of the 12 evaluated *IL6* SNPs, only rs10242595 was associated MD in the replication sample, with an estimated 10.6% increase in MD per A-allele (Table 5). In the pooled analysis with data from the Singapore Chinese women and the NBCSP, rs10242595 A-allele was associated with a 6.2% increase in MD (P = 0.0001).

**Table 5:** Association between IL6 SNPs and MD in Norwegian women, Singapore Chinese women, and the combined analysis including both populations.

	Norwegian^a^ Study						Singapore^b^ Study						Combined^c^		
SNP	Alleles	RA^d^	RAF e	beta	SE	P	Alleles	RA^e^	RAF	beta	SE	P	beta	SE	P
**rs10242595**	G/A	A	0.26	5.60	1.56	**0.0004**	A/G	A	0.96	10.65	4.62	**0.02**	6.18	1.50	**<.0001**
**rs12700386**	C/G	G	0.19	1.90	1.74	0.27	C/G	G	0.04	−2.83	4.79	0.56	1.27	1.66	0.44
**rs17147230**	T/A	A	0.01	2.09	9.37	0.82	A/T	A	0.53	2.15	1.91	0.26	2.14	1.80	0.23
**rs1880242**	G/T	T	0.49	3.84	1.37	0.0053	G/T	T	0.21	−3.45	2.40	0.15	1.87	1.20	0.12
**rs2056576**	C/T	T	0.29	1.33	1.51	0.38	C/T	T	0.03	−3.95	5.35	0.46	0.88	1.47	0.55
**rs2066992**	G/T	T	0.03	5.33	4.31	0.22	T/G	T	0.80	2.56	2.42	0.29	3.13	2.06	0.13
**rs2069837**	A/G	G	0.09	2.72	2.31	0.24	A/G	G	0.16	−0.78	2.63	0.77	1.11	1.72	0.52
**rs2069840**	C/G	G	0.33	3.40	1.41	0.02	C/G	G	0.03	−7.45	5.94	0.21	2.74	1.40	0.05
**rs4552807**	T/A	A	0.38	3.54	1.37	0.01	A/T	A	0.48	2.71	7.88	0.73	3.50	1.39	0.01
**rs6949149**	G/T	T	0.04	6.06	3.47	0.08	T/G	T	0.68	2.21	2.03	0.28	3.05	1.70	0.07
**rs6952003**	T/A	A	0.22	3.44	1.66	0.04	T/A	A	0.25	−2.41	2.16	0.27	1.10	1.32	0.40
**rs6969502**	G/A	A	0.15	0.99	1.79	0.58	A/G	A	0.68	2.47	2.02	0.22	1.67	1.33	0.21

a From linear regression models adjusting for age at mammogram (continuous) and BMI at mammogram (continuous). Additive genetic model was used.

b From linear regression models adjusting for age at mammogram (continuous) and BMI at mammogram (continuous) and dialect group (Cantonese, Hokkien). Additive genetic model was used. rs11766273, rs1880241, rs2069833 and rs7776857were excluded from the Singapore study because of low MAF.

c From linear regression models adjusting for age at mammogram (continuous) and BMI at mammogram (continuous) and ethnic and dialect group (Norwegian, Cantonese, Hokkien). Additive genetic model was used.

d Risk allele; the risk allele was defined based on the minor allele in the Norwegian sample.

e Riske allele frequency.

## Discussion

We studied the association between MD and the SNPs in 7 growth factor or cytokine genes including *IGFBP1*, *IGFBP3, TNF, FGFR2, VEGF, GHRHR*, and *IL6.* We observed statistically significant effect modification by menopausal status. While there were no significant associations for SNPs in 6 of the genes, 9 SNPs in the *IL6* region (rs6952003, rs10242595, rs11766273, rs1880241, rs1880242, rs2069833, rs2069840, rs4552807, rs7776857) were each significantly associated with MD in premenopausal women. MD varied between 3.4% to 5.8% per allele for these SNPs. Several of the associations were statistically significantly modified by BMI; the associations were limited to women with low BMI. The association with rs10242595 was replicated in an independent study of Singapore Chinese women. Given that each 1% increment in MD has been shown to be associated with a 2% higher relative risk of breast cancer [46], the magnitude of these associations suggest that these variants could be clinically significant.

The lack of association we found between SNPs in most of these growth factor and cytokine genes (*IGFBP1, IGFBP3, FGFR2, VEGF* and *GHRHR*) and MD is consistent with results from the few studies that have been conducted on these genes and MD. Consistent with our findings, the majority of previous studies investigating *IGFBP1* and *IGFBP3* SNPs reported a lack of significant association between *IGFBP1/IGFBP3* SNPs including rs2854746, rs1553009, rs1065780, rs2132570, rs3110697, rs35539615, rs4619, and rs6670 and MD. These studies include a cross-sectional study among 1,121 of premenopausal and postmenopausal women from the Nurses’ Health Study cohort investigating 13 tagging SNPs [27], a study of 819 pre- and postmenopausal women of Hawaiian, European, and Japanese ancestry from the Multiethnic Cohort study investigating 22 tagging SNPs [30], and a study of 1,916 premenopausal women within the Prospect-EPIC cohort investigating 11 tagging SNPs [47]. In the study by Tamimi et al., rs4619 in *IGFBP1/IGFBP3* region was positively associated with increased MD in a mixed population of premenopausal and postmenopausal women [27], however, this association was not observed in another study [30] nor in our study. Results from the Multiethnic Cohort study showed no association between *IGFBP1/IGFBP3* rs10228265, rs1496497 and rs3110697 and MD in the overall analysis, but a significant association was found when the analysis was limited to women with Hawaiian and Japanese descent [30]. In that study, the results were based on data from premenopausal and postmenopausal women pooled together. Our finding of no significant association between *FGFR2* rs2981582 and MD is consistent with a study of 516 white (429 non-Hispanic, 87 Hispanic) women in the age range of 20 to 49 years [48] and in a study of 825 pre-and postmenopausal women within the Multiethnic Cohort study [49]. We looked at only one SNP in *GHRHR* gene (rs4988496) and found no significant association. Similarly, in a study of 177 premenopausal women [50] a different polymorphism, *GHRHR* A57T, was reported as not significantly associated with MD.

While there have been no previous studies on *IL6* SNPs and MD, there are experimental data suggesting that our significant findings are biologically plausible. Cultures of normal mammary epithelial cells obtained from healthy women were shown to release interleukin-6 and express interleukin-6 receptor [51]. Data coming from in vitro studies supports the pleiotropic (having both tumor promoting and tumor-counteracting effects) nature of interleukin-6 in breast tissue [52]. It seems plausible that variations in the *IL6* gene could have effects on cell growth and alter MD and eventually breast cancer risk.

Another plausible way to explain the effects of interleukin-6 on MD is indirectly through estrogen. Interleukin-6 has an important role in regulating estrogen synthesis in normal and malignant breast tissues. The activities of aromatase, estradiol 17β-hydroxysteroid dehydrogenase and estrone sulfatase have been shown to be influenced by interleukin-6 in these tissues [53].

Mammographic density is inversely associated with the amount of fat tissue in the breast. It is possible that genetic factors could influence MD by influencing the amount of fat in the breast. *IL6* rs10242595-A allele was associated with decreased total body fat mass in one study where fat mass was measured with dual energy X-ray absorptiometry [54]. Our finding is consistent with this result; we found a significant positive association between this polymorphism and MD. *IL6* rs1880242 has been significantly associated with decreased risk of obstructive sleep apnea syndrome; obesity is a strong risk factor for this syndrome [55]. Consistent with results from this study, we found a significant positive association between this polymorphism and MD. However, this may suggest that the observed association between this SNP and MD is driven by its association with non-dense breast area rather than the absolute density. When we tested the association with absolute density for this IL-6 SNP, the observed association was similar to percent MD.

In this study we found both positive and inverse associations with different IL6 SNPs. To what extent the *IL-6* tagging SNPs modify IL-6 protein levels, and the direction of effect on protein levels is not yet clear. The negative association observed for some of these SNPs and MD does not necessarily represent a negative association between serum or tissue levels or function of *IL-6* and MD. Future studies with available breast tissue samples, blood samples and mammographic density measurements would allow us to explore whether any of these associations represent tissue specific effects. However, it would be a challenge to assemble a large enough group of healthy women with breast tissue samples for such investigations.

In this study the association between 5 *IL6* SNPs and MD was significantly modified by BMI (Table 4). Higher magnitude and more significant associations in women with the BMI of 25 or less, suggests that the role of *IL6* variants in predicting MD is less important in obese women.

There were several strengths of our study. The study sample was selected from a population based study conducted within a national screening program, and the population studied is ethnically homogeneous. Further, we used previously validated MD assessment techniques, and collected detailed information regarding key MD risk factors. We also replicated our findings in a different ethnic group. BMI was considered a potentially confounding factor in this study; we controlled for this variable in all the analyses presented. Many previous studies of MD combined pre- and postmenopausal women, which could mask any findings in premenopausal women. Our analysis conducted separately in premenopausal and postmenopausal women, may have helped to clarify results in premenopausal women. A limitation of our study was using buccal samples for genotyping of the NBCSP samples, which resulted in a relatively lower call-rate compared to the results from studies using blood samples. Further, given the nature of these screening programs, relatively few women were eligible for our study of premenopausal women. Finally, although it could have been more informative to examine the association between genetic variants, serum or tissue levels of growth factors/cytokines as intermediates, and MD, we did not have serum or tissue available to perform such analyses.

### Conclusions

Our study suggests that SNPs in the *IL6* region may be associated with MD in premenopausal women. Future studies should be conducted to relate these SNPs and interleukin-6 concentrations as well as *IL6* gene expression in the mammographically dense tissue to elucidate the mechanisms underlying this association.

## Supporting Information

Table S1 **Association between polymorphisms in growth-factor/cytokine pathway genes and MD by menopausal status in Norwegian women.**
(XLSX)

Table S2 **Association between polymorphisms in growth-factor/cytokine pathway genes and MD in Norwegian women (n = 301).**
(XLS)
